# Time frequency analysis of olfactory induced EEG-power change

**DOI:** 10.1371/journal.pone.0185596

**Published:** 2017-10-10

**Authors:** Valentin Alexander Schriever, Pengfei Han, Stefanie Weise, Franziska Hösel, Robert Pellegrino, Thomas Hummel

**Affiliations:** 1 Smell & Taste Clinic, Department of Otorhinolaryngology, Technical University of Dresden, Dresden, Germany; 2 Abteilung Neuropädiatrie Medizinische Fakultät Carl Gustav Carus, Technische Universität, Dresden, Germany; Université catholique de Louvain, BELGIUM

## Abstract

**Objectives:**

The objective of the present study was to investigate the usefulness of time-frequency analysis (TFA) of olfactory-induced EEG change with a low-cost, portable olfactometer in the clinical investigation of smell function.

**Materials & methods:**

A total of 78 volunteers participated. The study was composed of three parts where olfactory stimuli were presented using a custom-built olfactometer. Part I was designed to optimize the stimulus as well as the recording conditions. In part II EEG-power changes after olfactory/trigeminal stimulation were compared between healthy participants and patients with olfactory impairment. In Part III the test-retest reliability of the method was evaluated in healthy subjects.

**Results:**

Part I indicated that the most effective paradigm for stimulus presentation was cued stimulus, with an interstimulus interval of 18-20s at a stimulus duration of 1000ms with each stimulus quality presented 60 times in blocks of 20 stimuli each. In Part II we found that central processing of olfactory stimuli analyzed by TFA differed significantly between healthy controls and patients even when controlling for age. It was possible to reliably distinguish patients with olfactory impairment from healthy individuals at a high degree of accuracy (healthy controls vs anosmic patients: sensitivity 75%; specificity 89%). In addition we could show a good test-retest reliability of TFA of chemosensory induced EEG-power changes in Part III.

**Conclusions:**

Central processing of olfactory stimuli analyzed by TFA reliably distinguishes patients with olfactory impairment from healthy individuals at a high degree of accuracy. Importantly this can be achieved with a simple olfactometer.

## Introduction

Early research in the field of olfaction has focused on physiological evaluations using standard smell tests such as UPSIT and “Sniffin’ Sticks” while evaluating trigeminal functionality with menthol lateralization tests [1–3]. These procedures are fast, reliable and can segregate individuals into groups of differencing chemosensory abilities. Additionally, the temporal information of the same processes has been investigated, non-invasively with event-related potentials (ERP) [4]. This type of measurement became possible after the introduction of appropriate olfactory stimulus delivery devices that reduced somatosensory responses and provided chemosensory stimuli in a precise, timely matter [5]. The ERP technique has a high temporal resolution; and its response is directly related to the neuronal activation, which is time-locked to the stimulus onset. Since its inception in the olfactory field, numerous studies have used ERP to assess various dimensions of odors, demographic response differences, or differences in olfactory functionality. Typically, using across-trial averaging in the time-domain ERP can be characterized as a negative wave peaking approximately 320–500 ms after stimulus onset (N1), followed by a positive wave peaking approximately 450–800 ms after stimulus onset (P2 and/or P3) [6].

Latency and amplitude of chemosensory ERP, to date, have been relatively reproducible in normal individuals; however, its clinical usability in patients with olfactory impairments has been less effective due to the low signal-to-noise ratio [7, 8]. For instance, the presence of olfactory ERP always signifies the presence of olfactory function, but the absence of an ERP does not mean the opposite. Additionally, to obtain higher signal-to-noise chemosensory ERP more sophisticated olfactometers are needed that control temperature and present stimuli at higher flow (6–8 L / min, which requires humidified and thermostabilized air). These devices are expensive, bulky and may not be feasible for several clinics and research groups. Therefore, a low-cost, portable olfactometer may be more useful for clinical research of olfaction. In addition, a newer technique of analysis, the time-frequency analysis (TFA), has been proposed to increase the signal-to-noise ratio of chemosensory ERP responses [9]. Time-frequency examines the signal in both the time and frequency domains simultaneously by using, in our case a continuous wavelet transformation. This approach has been shown to increase the detectability of ERP, especially in response to olfactory stimuli, and allows for characterizing of non phase-locked components that could not be identified using conventional time-domain averaging in healthy and olfactory impaired individuals [9, 10]. Furthermore, these studies showed the magnitude of the ERP from TFA to significantly correlate with the psychophysical olfactory scores.

The objective of this study was to set up a clinical measurement of olfactory functions using a portable low-cost olfactometer, and to advance the understanding of using TFA in the chemosensory field. To accomplish this task, the study was composed of three parts on a custom built olfactometer. Part I was designed to optimize stimulus as well as recording conditions. In part II EEG-power change after olfactory/trigeminal stimulation was compared between healthy controls and patients with olfactory impairment. In Part III the test-retest reliability of the responses was evaluated in healthy subjects.

## Material and methods

All experiments were performed in accordance to the Declaration of Helsinki. The local Ethics Committee of the Medical Faculty of the TU Dresden approved the protocol (EK343092013). The test procedure was explained to and written consent was obtained from all participants.

### Part I: Optimizing the stimulus presentation

To test a simple method for objective olfactory evaluation, EEG-power change was recorded in response to olfactory as well as trigeminal stimuli.

#### Participants

A total of 40 healthy volunteers (20 women) aged from 18 to 33 years (mean age: 24±9 years) participated. All participants were normosmic as determined by means of the “Sniffin’ Sticks” test battery. The sample calculation (40 participants, 10 participants per stimulus condition) was based on the study done by Huart and colleagues, in which they used 11 healthy participants [9].

#### Stimuli and stimulus presentation

Three types of stimuli were used in this study: 1) phenyl-ethyl-alcohol (PEA–a rose like odor; Sigma, Deisenhofen, Germany) for olfactory stimulation, 2) eucalyptol (has a cooling effect and produces a burning sensation; Sigma, Deisenhofen, Germany) for mixed olfactory/trigeminal stimulation (from now on referred to as “trigeminal”), and 3) water as a control. A trigeminal stimulus was included in this study to ensure functioning of the new method. EEG changes e.g. ERP analyzed using the time-domain averaging method are more robust to trigeminal than to olfactory stimuli in healthy participants as well as in patients with olfactory impairment.

**Fig 1:**
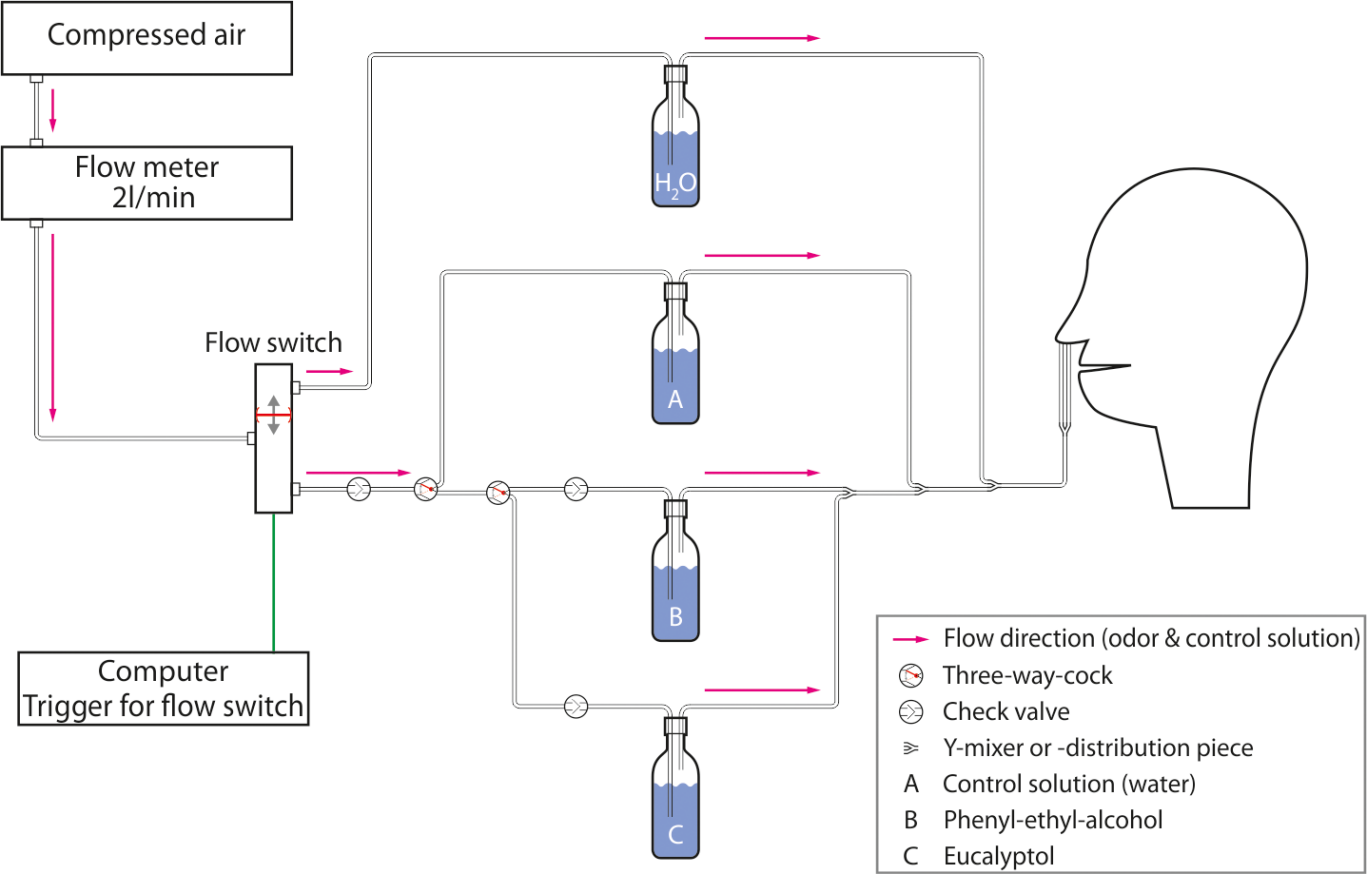
Olfactometer. Displayed is a schematic drawing of the custom-built olfactometer, which was used in the study. The flow switch was controlled by a computer directing the air either through a glass bottle with water (between stimuli) or through one of the bottles marked A, B or C for stimulus presentation. The stimuli were therefore presented in a clean–odorless airflow of 2l/min.

Stimuli were presented birhinally using a custom-built olfactometer (see Fig 1 for schematics). This olfactometer was chosen since it references an economical option for stimulus presentation. The airflow of the olfactometer was set to 2 L/min while chemosensory stimuli, either 500 to 1000ms in duration, were embedded between varying interstimulus interval (ISI) of clean air (12, 20, or randomized 18–20 s). Additionally, stimuli were either cued or not depending on the condition. To cue participants for a stimulus, the screen used for the tracking task (see *data acquisition* section) changed from a black to a red screen; however, this change was not locked to the stimulus onset and varied 1000 to 3000ms before the stimulus. Four different stimulus conditions (see Table 1) were tested for each stimulus (60 recordings per stimulus condition). The number of stimuli used in this study was based on previous work about olfactory ERP [8].

**Table 1:** Stimulus conditions used in part I.

Condition	A	B	C	D
**PEA concentration**	100%			
**Eucalyptol concentration**	1:40	1:7.5		
**Stimulus duration in s**	0.5	1		
**ISI in s**	12	20	18–20 randomized	
**Cued stimuli**	No			Yes
**Sessions**	3 sessions (PEA, eucalyptol, control) 60 stimuli each		Each stimulus 60 times, randomized and divided into 3 sessions	

#### Data acquisition

EEG was recorded with a 16-channel amplifier but only three scalp electrodes (Fz, Cz, Pz) were used according to the international 10–20 system [11] with linked earlobes (A1 –A2) as reference (16-channel amplifier; SIR: Röttenbach, Germany). EEG-segments lengths of 4000ms, starting 1000ms before stimulus onset, were recorded at a sampling frequency of 250Hz using a band-pass filter of 0.2-30Hz. To ensure their attention and preventing fast eye movements and blinking, the participants were asked to perform a computer task during the measurements, during which they had to keep a marker in a slowly moving square using the computer mouse. Additionally, participants were acoustically shielded by headphones with white noise (~ 60dB) to mask clicking sounds of the olfactometer.

### Part II: Evaluation in patients with olfactory dysfunction

The aim of Part II was to evaluate whether it is possible to objectively identify patients with olfactory dysfunction based on EEG-power changes after olfactory and trigeminal stimulation.

#### Participants

Twenty healthy participants (11 women) with a normal sense of smell aged from 20 to 30 years (mean 23.8±2.8 years) participated in this part. Similar to Part I, these participants had no diseases related to olfactory loss and scored in the normal olfactory functionality range measured by means of the “Sniffin’ Sticks” (mean 36.5±2.9 points) with a range of 32.0 to 41.5 points. Additionally, 18 patients (12 women) with olfactory dysfunction aged 20 to 68 years (mean 46.6±6.3 years) were included in this part of the study. Nine patients were classified as hyposmic and the other nine were regarded as functionally anosmic. Causes for olfactory dysfunction were: post-infectious (n = 4), medication (n = 1), post-traumatic (n = 8) and idiopathic (n = 5). Controls and patients significantly differed in age with controls being younger (t = 5.73, *p*<0.001), and patients scored significantly lower on the olfactory test (t = 10.96, *p*<0.001).

#### Stimuli and stimulus presentation

Based on Part I, only the settings of condition D were used for all stimuli since it produced the best results for olfactory stimulation. For condition D, stimuli were cued and presented with an ISI between 28-30s (randomized). The stimulus length was 1000ms and concentrations differed by stimulus type (PEA at 1:1 and eucalyptol at 1:7.5). Stimuli were randomly presented 60 times each over 3 sessions. There was a five-minute break between the sessions.

### Part III: Test-retest reliability

To evaluate the test-retest reliability of the results from TFA, 20 healthy participants from Part II were retested using olfactory stimuli with the same stimulus and recording conditions as in part II. The mean duration between the first and second testing varied by participants’ availability and was 12.5±13.6 days (range: 2 to 61 days).

### Data analysis

Pre-processing and analysis of EEG data were carried out using the Letswave 5 toolbox (http://nocions.webnode.com/letswave). The EEG data was filtered offline with a band-pass filter (FFT) of 0.1–15 Hz prior to segmentation of -1000 to 3000ms relative to stimulus onset. This was followed by a baseline correction with a reference interval of 1000ms before stimulus onset. Epochs containing artifacts (due to blinking or movement) were manually excluded following examination of the Fp2 electrode. In addition, recordings exceeding -50/50µV were rejected. The mean number of artifact free epochs was 29.6±19.9 (range 8–53).

TFA using the continuous Morlet wavelet transform (CWT) was applied. The Morlet wavelet consists of a complex exponential function localized in time by a Gaussian envelope. Similar to other studies, the initial spread of the Gaussian wavelet was set at 2.5/πω0 (ω0 being the central frequency of the wavelet) [9]. The time-frequency transform was first applied to single EEG epochs (CWT-SINGLE) with the frequency band set to 1-15Hz, and then single-trial time-frequency maps were averaged for each subject and stimulus type.

The CWT-SINGLE time-frequency maps were expressed relative to baseline (pre-stimulus interval ranging from 1000ms to 0ms relative to stimulus onset), as follows: ER% _t,f_ = (A t,f—R_f_)/R_f_, where A_t,f_ is the signal amplitude at a given latency t and frequency f, and R_f_ is the signal amplitude at the frequency f, averaged within the pre-stimulus reference interval. To detect EEG-power changes due to olfactory and trigeminal stimulation a region of interest (ROI) was defined: 200 – 2000ms after stimulus onset and in the frequency range 2 to 6 Hz (compare: [9]. The latency of the ROI was chosen fairly long (until 2000ms after stimulus onset) due to the low flow rate of 2l/min) of stimulus presentation. The average EEG-power change within the ROI compared to the pre-stimulus baseline mentioned above was calculated. Analysis was conducted and compared among all three recording electrodes (Cz, Pz and Fz) and the position with the largest EEG-power change and the largest AUC was chosen for further analyses (Part II and Part III).

### Statistical analyses

Data were analyzed by means of SPSS 22.0 (SPSS Inc., Chicago, IL, USA). T-tests were used to compare average EEG-power changes among conditions in Part I. To determine the sensitivity and specificity of distinguishing between the control and olfactory stimulus in Part I, and between normosmic participants and dysosmic patients in Part II, receiver-operating characteristic (ROC) analysis was conducted and the Youden-Index was calculated to obtain the largest sensitivity and specificity. Additionally for Part II, Pearson’s correlation analysis was used to determine EEG-power changes in relation to olfactory functionality among individuals while correlation analysis was used in Part III to obtain a measure of reliability. The level of significance was set at 0.05.

## Results

### Part I: Optimizing the stimulus conditions

**Fig 2:**
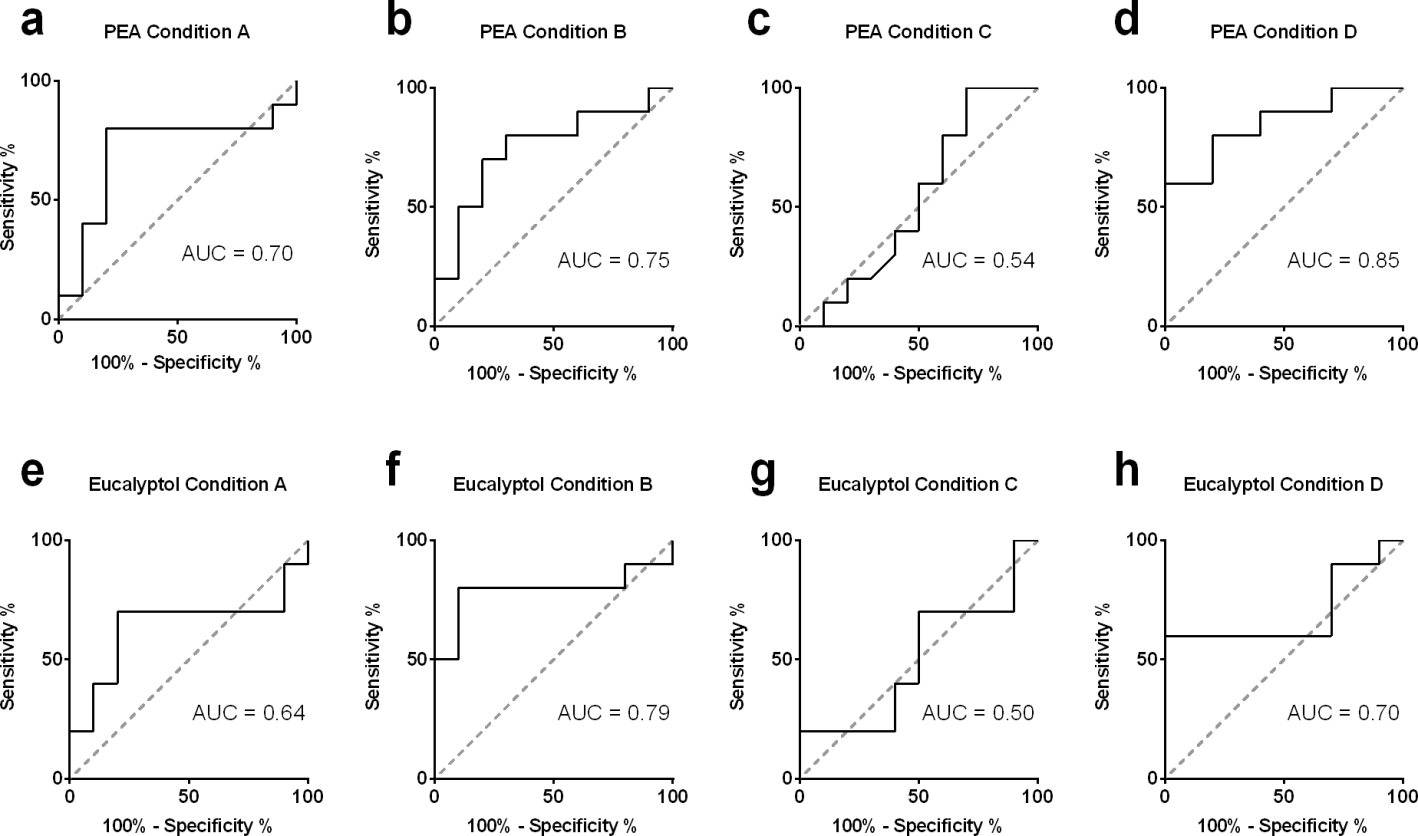
AUC in relation to different stimulus conditions. Displayed are the AUC calculated by ROC, a-d for olfactory and e-h for trigeminal stimulation for the four stimulus conditions. The largest AUC and therefore the highest sensitivity and specificity to distinguish between control and olfactory stimulation in terms of EEG-power change was obtained in condition D. The AUC analysis revealed only a significant AUC for trigeminal stimulation in condition B.

EEG-power changes in response to olfactory or trigeminal stimuli were obtained as shown in Table 2 and Table 3, separately. EEG-power changes for olfactory stimuli were highest for condition D for all electrodes (Table 2). In addition, the difference between olfactory-induced EEG-power change and control was significantly different for all three recording electrodes with condition D (p<0.05, Table 2). For condition D, ROC analysis showed that the area under the curve (AUC) was significant for all three electrodes (Cz: AUC = 0.85, p = 0.008; Fz: AUC = 0.81, p = 0.02; Pz: AUC = 0.81, p = 0.02). The largest stimulus-to-control ratio and the largest AUC were obtained from recording electrode Cz. Therefore, condition D was chosen for stimuli and Cz was chosen as the recording position for further analyses. When condition D was used to deliver the olfactory stimulus and EEG was recorded from electrode Cz it was possible to distinguish between the olfactory stimulus and control stimulus in terms of EEG-power changes with a sensitivity of 60% and a specificity of 100% when a cut off of 8.48% in EEG-power changes within the ROI was used (highest Youden-index) (see Fig 2).

**Table 2:** EEG-power in response to olfactory stimulation.

	Cz			Fz			Pz		
	PEA	Control	P	PEA	Control	p	PEA	Control	p
Condition A	5.51±7.02	6.80±3.25	0.67	5.47±11.07	6.34±5.10	0.79	4.57±5.54	3.33±5.02	0.64
Condition B	6.14±5.29	1.27±4.05	0.06	4.68±7.75	-0.41±6.26	0.19	7.80±9.57	3.68±7.67	0.21
Condition C	8.69±3.45	7.29±5.86	0.50	7.65±4.54	2.72±3.90	0.002	7.00±6.69	4.70±7.46	0.42
Condition D	**10.39±7.16**	**2.59±3.50**	**0.002**	9.80±6.53	3.71±3.89	0.04	11.43±7.82	3.15±4.62	0.004

Displayed are mean EEG-power changes in percent (±SD).

**Table 3:** EEG-power change in response to trigeminal stimulation.

	Cz			Fz			Pz		
	Eucalyptol	Control	P	Eucalyptol	Control	P	Eucalyptol	Control	P
Condition A	7.40±6.06	6.80±3.25	0.82	3.27±8.70	6.34±5.10	0.32	7.42±6.40	3.33±5.02	0.23
Condition B	10.82±10.45	1.27±4.05	0.02	13.07±11.35	-0.41±6.26	0.03	11.27±10.42	3.68±7.67	0.13
Condition C	8.78±9.57	7.29±5.86	0.66	8.80±7.22	2.72±3.90	0.03	8.44±9.94	4.70±7.46	0.07
Condition D	**8.46±7.99**	**2.59±3.50**	**0.02**	9.98±9.16	3.71±3.89	0.06	9.05±7.81	3.15±4.62	0.08

Displayed are mean EEG-power changes in percent (±SD).

Table 3 shows that EEG-power changes for the trigeminal stimuli (eucalyptus). The highest EEG-power change was found in condition B. The stimulus parameters used in condition D also lead to a significant EEG-power change after trigeminal stimulation. The focus of this study was to evaluate an easy to use objective method to distinguish between normosmia and olfactory dysfunction. With this in mind and to keep consistency with olfactory stimulation, condition D was also chosen as stimulus condition for part II in regard to trigeminal stimulation. However, the ROC analyses showed no significant AUC for condition D for all three recording electrodes (Cz: AUC = 0.70, p = 0.13; Fz: AUC = 0.72, p = 0.10; Pz: AUC = 0.74, p = 0.07), meaning that it was not possible to distinguish between trigeminal and control condition by means of EEG-power change.

**Fig 3:**
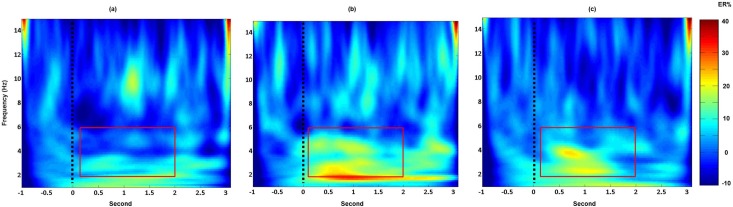
EEG-power change after olfactory, trigeminal and control stimulation (condition D). Displayed are the Cz EEG-power changes (group average including the 10 participants) after control (a), olfactory (b) and trigeminal (c) stimulation. The dashed black line marks the stimulus onset. An increase in low frequency within the ROI (red square) can be observed in b and c but not in a. The scale displays EEG-power change in percent compared to the pre-stimulus interval.

Fig 3 displays the EEG-power change after olfactory/trigeminal and control stimulus with condition D. As shown, after olfactory and trigeminal stimulation an increase in EEG-power within the ROI can be observed (Fig 3).

Based on these results stimulus condition D was used in study Parts II and III.

### Part II: Evaluation in patients with olfactory dysfunction

**Fig 4:**
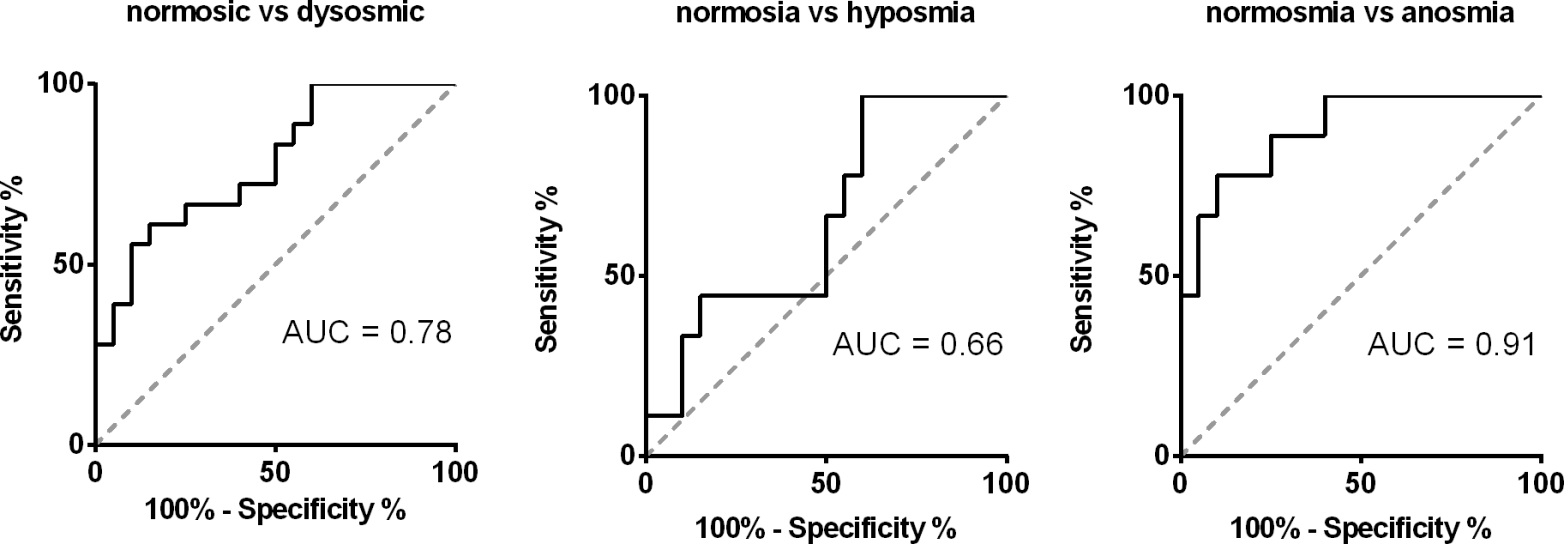
Distinguishing between patients and normosmic participants by means of time-frequency analysis. Displayed are the AUC calculated by ROC analysis to distinguish between normosmic participants and dysosmic (hyposmic/anosmic) patients. The analysis revealed an AUC of 0.78 for the separation of normosmic participants and dysosmic patients. The analysis between normosmic participants and anosmic patients was even greater with an AUC of 0.91.

Normosmic participants had a significantly higher EEG-power change after olfactory stimulation with PEA compared to patients (6.91±8.60% vs -1.18±7.41%. t = 3.25, p = 0.002), however, the difference was no longer significant when controlling for age (F = 1.95, p = 0.17) When comparing to anosmic patients, healthy controls had significantly larger EEG-power change after PEA stimulation (6.91±8.60% vs -5.46±5.57%. t = 3.94, p = 0.001). This was still true when controlling for age (F = 4.94, p = 0.035). However, the comparison between normosmic and hyposmic people missed significance (6.91±8.60% vs 2.23±7.27%. t = 1.42, p = 0.17 without age correction; and F = 0.23, p = 0.64 corrected for age). In addition, after controlling for age, a positive correlation between EEG-power change due to olfactory stimulation and olfactory test score was found for odor discrimination, identification and whole TDI (discrimination: r = 0.351, p = 0.036; Identification: r = 0.443, p = 0.007; TDI:,r = 0.424, p = 0.010), and also a trend for odor threshold was found (r = 0.36, p = 0.059). ROC analysis revealed an AUC of 0.78 when normosmic participants were compared to patients, and by choosing a cut-off of 2,1% in EEG-power change (determined by the highest Youden-index), it was possible to distinguish between participants and patients with a sensitivity of 75% and a specificity of 67%. Furthermore, comparing anosmics to normosmics the AUC was even higher at 0.91, and by using the same cut of as above (2.1% in EEG-power change) a sensitivity of 75% and a specificity of 89% was found. However it was not possible to distinguish between normosmic participants and hyposmic patients by means of EEG-power change using the ROC analysis. Fig 4 displays the results of the AUC analysis by ROC.

For trigeminal stimulation, no significant difference in EEG-power changes existed between normosmic participants and patients (mean EEG-power change within the ROI in normosmic participants and patients: 9.32±8.54% vs 7.20±12.60%; t = 0.60, p = 0.55; and F = 0.58, p = 0.45 after controlling for age). Furthermore, when patients were divided into hyposmics and anosmics the results did not differ significantly from EEG-power changes observed in normosmics after trigeminal stimulation (p > 0.10). No correlation between EEG-power change and olfactory test scores (TDI) was observed (r = 0.296, p = 0.13), and it was not possible to distinguish between normosmic participants and patients by means of ROC analysis in terms of EEG-power change after trigeminal stimulation (AUC = 0.59, p = 0.36).

### Part III: Test-retest reliability

EEG-power change after olfactory stimulation was significantly correlated between the test retest sessions after controlling for age (r = 0.460, p = 0.048).

## Discussion

Analysis of olfactory induced EEG-power changes by means of TFA allows to distinguish between patients with olfactory dysfunction and normosmic individuals with higher precision than previously possible [9, 10]. Our study provides additional information to the clinical usefulness of central odor processing by means of TFA to distinguish between patients with olfactory dysfunction and normosmic individuals. In addition we were able to show that a rather easy and cost efficient custom-built olfactometer suffices to answer the question whether there is olfactory processing or not.

Findings of our study show that the optimal stimulus duration was 1000ms with ISIs of 18-20s for both olfactory and trigeminal stimuli. In addition better results were obtained with cued compared to non-cued stimuli, possibly due to an increased attention among individuals. In our study, this setup showed a significant difference over the control with an EEG-power response difference of 10.39% for olfactory and 8.46% for trigeminal stimulation. Previous work on olfactory ERP has shown that ERP Amplitudes due to attended olfactory stimuli are larger compared to unattended stimuli [12]. By obtaining the best results in the cued stimulus condition D we could show that this is also true if TFA is used for analyzing central odor processing.

A stimulus length of 200-400ms was commonly used to examine central odor processing by means of EEG [13–15]. In our study we used stimulus durations of 500ms to 1000ms. A stimulus duration of 1000ms resulted in better results compared to a stimulus lengths of 500ms. This finding is most likely caused by the larger number of molecules that reach the olfactory cleft when the stimulus duration is increased. That is, while an increase of stimulus duration is not so important for olfactory ERP because this is mostly due to the steepness of stimulus onset [16], for TFA it is, since the entire EEG segment is influenced by the longer stimulus duration and so is the result of TFA. More than one stimulus parameter was changed between stimulus conditions. Therefore it is only possible to compare the four used stimulus conditions rather than the individual stimulus parameters.

Taking this optimal stimulus presentation, EEG-power changes after olfactory stimulation calculated by TFA could reliably distinguish patients with olfactory impairment from healthy individuals at a high degree of accuracy. Furthermore breaking down patients into two olfactory impairment groups it showed that this was driven by the comparison between anosmic patients and healthy participants. It was not possible to distinguish between normosmics and hyposmic patients by means of EEG-power change. Overall a correlation of EEG-power change and results of psychophysical olfactory testing was observed. This is in line with results reported previously by Huart and colleagues [9]. Meanwhile, no difference in EEG-power changes after trigeminal stimulation was shown between patients and healthy controls. This is in line with previous studies, which have shown that the trigeminal sensitivity is usually intact for individuals with impaired olfaction [10]. It has to be pointed out that trigeminal stimulation lead to higher EEG-power change in the current study, but that the ROC analysis did not show significant results when comparing EEG-power change after trigeminal and control stimulation. This could be due to the custom-built olfactometer. With this simple device a trigeminal stimulation in the control condition resulting from air-flow changes when opening and closing a valve, can not be excluded. Further studies are needed to optimize the stimulus and conditions to study EEG-power change after trigeminal stimulation. The focus of this study was on central olfactory processing.

Our findings are in line with those of Huart and colleagues who were able to distinguish dysosmic patients from normosmic people by means of ERP analyzed by TFA using common stimulus parameters suggested by Evans et al. (AUC 0.88, sensitivity 91%, specificity 86%)[10, 14]. Furthermore, our study adds to the findings of Huart and colleagues with a slightly larger sample size (33 vs. 38) and the use of a more economical olfactometer. To the later point, our results reveal the possibility of acquiring an electrophysiological, EEG-derived parameter without the use of an expensive olfactometer [10]. Such an easy to use olfactometer for chemosensory presentation could also open the door for more research groups that aim to understand the cortical processes involved in the perception of odors. However, more research is needed to improve the mechanical features of the olfactometer, and to optimize the stimulus parameters, in order to obtain a more effective determination of olfactory functions with higher sensitivity and specificity for clinical purposes.

We observed an effect of age on the EEG-power change after olfactory stimulation in the current study. This is in line with previous work on olfactory ERP that reported longer latencies and reduced amplitudes in old compared to young people [17, 18]. Even though the control group and patient population differed significantly in terms of age in our study EEG-power change was significantly larger in normosmic compared to anosmic patients when controlling for age.

The test-retest reliability of TFA of chemosensory induced EEG-power change was significant for PEA. Welge-Lussen and colleagues reported correlation coefficients of 0.4 to 0.75 regarding olfactory ERP parameters using the time-domain analysis [19]. Our results are significant and show the reliability of this method but is compared to previous work on the lower end of the scale regarding the correlation coefficients. There are several reasons, which may explain this situation. First, the sample for this sub-analysis was relatively small (n<20) and homogeneous (only young, healthy subjects). Because correlative analyses are based on variance a future study needs to include more subjects with different ages or degrees of olfactory function. Second, current results show that EEG-power changes during the first session were much greater than during the second. This is likely to be due to the nature of the experiments where the first contact is always more exciting and salient than a second encounter with the same stimuli and conditions. So it can be assumed that by further optimizing the conditions a even better rest retest reliability will result. Yet, this needs to be investigated further in future studies.

## Conclusion

The study demonstrated the use of a low-cost, portable olfactometer, and the TFA to study the central processing of olfactory stimuli. The response change can reliably distinguish patients with olfactory impairment from healthy individuals at a high degree of accuracy.
